# Expert consensus on orthodontic treatment of patients with periodontal disease

**DOI:** 10.1038/s41368-025-00356-w

**Published:** 2025-04-03

**Authors:** Wenjie Zhong, Chenchen Zhou, Yuanyuan Yin, Ge Feng, Zhihe Zhao, Yaping Pan, Yuxing Bai, Zuolin Jin, Yan Xu, Bing Fang, Yi Liu, Hong He, Faming Chen, Weiran Li, Shaohua Ge, Ang Li, Yi Ding, Lili Chen, Fuhua Yan, Jinlin Song

**Affiliations:** 1 College of Stomatology, Chongqing Medical University, Chongqing Key Laboratory of Oral Diseases, Chongqing Municipal Key Laboratory of Oral Biomedical Engineering of Higher Education, Chongqing Municipal Health Commission Key Laboratory of Oral Biomedical Engineering, Chongqing, China; 2 State Key Laboratory of Oral Diseases, National Clinical Research Center for Oral Diseases, West China Hospital of Stomatology, Sichuan University, Chengdu, China; 3 Department of Pediatric Dentistry, West China Hospital of Stomatology, Sichuan University, Chengdu, China; 4 Department of Periodontics, Liaoning Provincial Key Laboratory of Oral Diseases, School and Hospital of Stomatology, China Medical University, Shenyang, China; 5 Department of Orthodontics, Beijing Stomatological Hospital, Capital Medical University, Beijing, China; 6 State Key Laboratory of Military Stomatology and National Clinical Research Center for Oral Diseases and Shaanxi Clinical Research Center for Oral Diseases, Department of Orthodontics, School of Stomatology, Air Force Medical University, Xi’an, China; 7 Jiangsu Key Laboratory of Oral Diseases, Nanjing Medical University, Nanjing, China; 8 Department of Periodontics, Affiliated Hospital of Stomatology, Nanjing Medical University, Nanjing, China; 9 Department of Orthodontics, Shanghai Ninth People’s Hospital, Shanghai Jiao Tong University School of Medicine, Shanghai Jiao Tong University, Shanghai, China; 10 Laboratory of Tissue Regeneration and Immunology and Department of Periodontics, Beijing Key Laboratory of Tooth Regeneration and Function Reconstruction, School of Stomatology, Capital Medical University, Beijing, China; 11 Immunology Research Center for Oral and Systemic Health, Beijing Friendship Hospital, Capital Medical University, Beijing, China; 12 State Key Laboratory of Oral & Maxillofacial Reconstruction and Regeneration, Key Laboratory of Oral Biomedicine Ministry of Education, Hubei Key Laboratory of Stomatology, School & Hospital of Stomatology, Wuhan University, Wuhan, China; 13 Department of Orthodontics, School & Hospital of Stomatology, Wuhan University, Wuhan, China; 14 State Key Laboratory of Oral & Maxillofacial Reconstruction and Regeneration, National Clinical Research Center for Oral Diseases, Shaanxi International Joint Research Center for Oral Diseases, Department of Periodontology, School of Stomatology, The Fourth Military Medical University, Xi’an, China; 15 Department of Orthodontics, Peking University School and Hospital of Stomatology, Beijing, China; 16 Department of Periodontology, School and Hospital of Stomatology, Cheeloo College of Medicine, Shandong University and Shandong Key Laboratory of Oral Tissue Regeneration and Shandong Engineering Research Center of Dental Materials and Oral Tissue Regeneration and Shandong Provincial Clinical Research Center for Oral Diseases, Jinan, China; 17 Key Laboratory of Shaanxi Province for Craniofacial Precision Medicine Research, College of Stomatology, Xi’an Jiaotong University, Xi’an, Shaanxi China; 18 Department of Periodontology, College of Stomatology, Xi’an Jiaotong University, Xi’an, Shaanxi China; 19 State Key Laboratory of Oral Diseases & National Center for Stomatology & National Clinical Research Center for Oral Diseases, West China Hospital of Stomatology, Sichuan University, Chengdu, China; 20 Department of Periodontics, West China Hospital of Stomatology, Sichuan University, Chengdu, China; 21 Hospital of Stomatology, Guanghua School of Stomatology, Sun Yat-sen University, Guangzhou, China; 22 Guangdong Provincial Key Laboratory of Stomatology, Guangzhou, China; 23 Department of Periodontology, Nanjing Stomatological Hospital, Affiliated Hospital of Medical School, Institute of Stomatology, Nanjing University, Nanjing, China

**Keywords:** Health care, Diseases

## Abstract

Patients with periodontal disease often require combined periodontal-orthodontic interventions to restore periodontal health, function, and aesthetics, ensuring both patient satisfaction and long-term stability. Managing these patients involving orthodontic tooth movement can be particularly challenging due to compromised periodontal soft and hard tissues, especially in severe cases. Therefore, close collaboration between orthodontists and periodontists for comprehensive diagnosis and sequential treatment, along with diligent patient compliance throughout the entire process, is crucial for achieving favorable treatment outcomes. Moreover, long-term orthodontic retention and periodontal follow-up are essential to sustain treatment success. This expert consensus, informed by the latest clinical research and practical experience, addresses clinical considerations for orthodontic treatment of periodontal patients, delineating indications, objectives, procedures, and principles with the aim of providing clear and practical guidance for clinical practitioners.

## Introduction

Periodontal diseases are common chronic inflammatory conditions affecting the supportive tissues of the teeth (gingiva, alveolar bone, and periodontal ligament), predominantly presenting as gingivitis and periodontitis.^1,2^ Gingivitis, characterized by reversible inflammation of the gingiva, can occur at any age, resulting in gingival swelling and bleeding on probing (BOP). Periodontitis involves the progressive destruction of periodontal tissues, afflicting over half of adults. Main symptoms of periodontitis include gingival bleeding and inflammation, formation of periodontal pockets, alveolar bone loss, and tooth mobility and migration.^3^ Progressive deterioration of periodontal tissues may lead to drifting and flaring of teeth, secondary occlusal trauma, bite collapse, and ultimately tooth loss, impairing masticatory function, aesthetics, and quality of life.^4^

Individuals seeking orthodontic treatment predominantly consist of children and adolescents. In recent years, the rising interest in dental aesthetics coupled with the increasing prevalence of periodontitis and its associated secondary malocclusions have led to a growing demand among adults for orthodontic intervention.^5–7^ Consequently, orthodontists are now encountering more complex adult cases with periodontitis.^5^ This shift highlights not only the importance of periodontal therapy in orthodontic treatment for periodontal health but also underscores the integration of orthodontic tooth movement into periodontal treatment to achieve stable occlusion, particularly in cases involving secondary malocclusions. This trend promotes the development of an integrated periodontal-orthodontic treatment approach.^4,8–17^

Periodontal-orthodontic combined therapy offers comprehensive orthodontic treatment for patients with periodontal disease following effective periodontal therapy. The core of this approach involves moving teeth to optimize tooth alignment and adjust occlusal relationship, thereby facilitating oral hygiene maintenance, improving occlusal function, and enhancing aesthetics. Recent systematic reviews have highlighted improved clinical outcomes in periodontitis patients treated with this integrated therapy.^12,18,19^ Additionally, long-term studies with follow-up periods of up to 10 years have demonstrated the durability and stability of the therapeutic outcomes.^20–23^ Therefore, understanding the principles of integrating orthodontics into periodontal management is essential for clinicians to better serve these patients.

This expert consensus focuses on clinical considerations for orthodontic treatment of patients with periodontal disease, delineating treatment indications, goals, procedures, and principles. By integrating the latest clinical research and practical experience, it provides comprehensive guidance for periodontal-orthodontic therapy, aiming to optimize treatment results while upholding treatment safety and effectiveness. We hope this consensus offers a clear, practical reference framework for clinicians, promoting standardization and consistency in orthodontic treatment for periodontal patients.

## Indications And Goals Of Orthodontic Treatment For Patients With Periodontal Disease

### Indications

Orthodontic treatment is indicated for patients with periodontal disease in the following scenarios:

- For patients with gingivitis or mild periodontitis who wish to undergo orthodontic treatment, therapy can begin following standard periodontal treatment.
- When pre-existing malocclusion contributes to periodontal tissue damage, such as gingival trauma caused by deep overbites or impaired cleaning due to crowding, orthodontic treatment can promote periodontal health.
- In cases of moderate to severe periodontitis, secondary malocclusion may arise due to reduced periodontal support, resulting in pathological tooth migration triggered by factors such as chewing forces, tongue movement, and lip pressure. In these instances, orthodontic treatment in conjunction with periodontal therapy becomes necessary to alleviate occlusal trauma and promote periodontal healing.
- For patients with periodontitis seeking aesthetic or restorative improvements, orthodontic treatment can address concerns such as reducing interproximal spaces, adjusting gingival margin height, correcting tilted molars, or creating space for restorative procedures.

### Goals

For patients with periodontal disease, the goals of orthodontic treatment include:

- Facilitating periodontal maintenance: Correcting misaligned teeth and relieving crowding help reduce areas where plaque accumulates, which facilitates personal and professional oral hygiene.
- Establishing a stable functional occlusion: Orthodontic treatment can create a favorable biomechanical environment for the healing of affected periodontal tissues by removing traumatic occlusion, reducing overbite and overjet for proper anterior guidance, and establishing balanced posterior support. In cases of missing teeth, orthodontic treatment can also create space for restoration, promoting long-term occlusal stability.
- Achieving improved aesthetics: Orthodontic treatment can close diastema, retract flared anterior teeth, and improve gingival margins, thereby enhancing smile aesthetics. This is a key motivator for many patients with periodontitis seeking orthodontic treatment.

## Periodontal-Orthodontic Treatment Sequence

Recently, the European Federation of Periodontology (EFP) has released S3-level clinical practice guidelines for managing periodontal disease.^24^ These guidelines advocate a stepwise approach to therapy, tailored to the disease stage. The first step prioritizes improving oral hygiene and addressing risk factors, which involves oral hygiene instructions, removal of supragingival plaques and calculus, and behavioral interventions (e.g., for smoking cessation and diabetes management). The second step targets reducing or eliminating subgingival biofilm and calculus through subgingival instrumentation, potentially supplemented with chlorhexidine or antimicrobials in a short time. The third step addresses areas exhibiting inadequate response to step two (pockets ≥ 5 mm with BOP, or pockets ≥ 6 mm). This stage includes surgical interventions such as non-regenerative surgeries (e.g., access flap surgery and resective surgery) to facilitate further subgingival instrumentation, or regenerative surgeries to restore periodontal soft or bone tissues. Supportive periodontal care (SPC) aims to maintain periodontal health in all treated patients through regular follow-up visits and re-treatment when necessary.

**Fig. 1:**
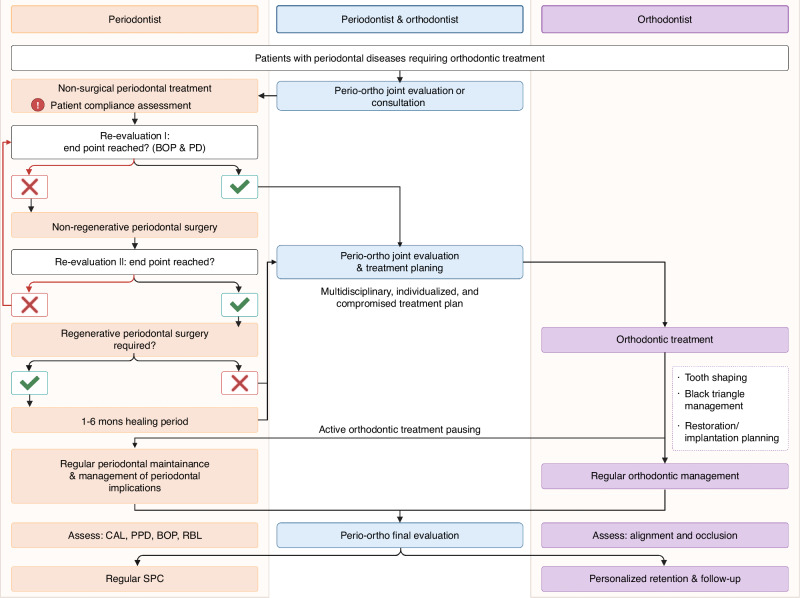
Flowchart illustrating the integration of orthodontic treatment into periodontal management for patients with periodontal disease (modified version; originally sourced from Papageorgiou et al. ^12^). Perio-ortho, Periodontal-orthodontic; BOP, bleeding on probing; PPD, probing pocket depth; CAL, clinical attachment loss; RBL, radiographic bone loss; SPC, supportive periodontal care

For patients with gingivitis, orthodontic interventions may commence following the first step of periodontal therapy. In cases of periodontitis featuring pathogenic tooth migration, missing teeth, or occlusal trauma, orthodontic treatment should be planned from the beginning and implemented after or concurrently with standard periodontal therapy. Detailed protocols and strategies for combined periodontal and orthodontic interventions for patients with periodontal disease are delineated in this section (Fig. 1).

### Periodontal treatment

#### Perio-ortho joint evaluation/consultant

After the initial examination, an interdisciplinary consultation between the periodontist and orthodontist is beneficial for preliminary discussions regarding personalized treatment procedures and patient-specific considerations. The prognoses of teeth as either hopeless, questionable, or safe need to be assessed since compromised teeth often serve a beneficial purpose during orthodontic treatment, such as providing anchorage reinforcement or augmenting alveolar bone with orthodontic extrusion. For patients with risk factors such as smoking or diabetes, interventions to educate and advice patients for behavioral changes (e.g., smoking cessation, improved metabolic control of diabetes) are necessary to facilitate control of periodontitis.

#### Non-surgical periodontal treatment

Non-surgical periodontal treatment involves the professional removal or reduction of supra and subgingival plaque and dental calculus (with or without adjunctive therapies), providing oral hygiene instructions, managing risk factors, and enhancing patient compliance.

#### Re-evaluation I

The assessment of individual responses to non-surgical periodontal treatment should be conducted following an appropriate healing period.^25^ If the endpoints of periodontal therapy (no periodontal pockets ≥ 5 mm with BOP or no deep pockets ≥ 6 mm) have been achieved, patients may proceed with orthodontic treatment after perio-ortho joint treatment planning. However, patients afflicted with severe periodontitis may fail to attain these endpoints due to the presence of deep pockets or intricate anatomical features, necessitating further periodontal surgery. Moreover, meticulous evaluation of patient compliance is essential, as the EFP advises against undertaking periodontal surgery in individuals failing to attain adequate levels of self-performed oral hygiene.^24^

#### Non-regenerative surgery

Non-regenerative surgical procedures include flap surgery and resective periodontal surgery to benefit self-oral hygiene control and improve access for subgingival instrumentation. In cases of deep residual pockets (≥ 6 mm) after an adequate non-surgical periodontal treatment, the EFP suggests using resective surgery compared with flap surgery, but an issue of gingiva recession needs to be considered.^24^

#### Re-evaluation II

If the endpoints have been achieved following non-regenerative surgery, orthodontic treatment may proceed after perio-ortho joint treatment planning for patients who do not require periodontal regeneration. In cases with intrabony defects or furcation involvement, periodontal regenerative surgery becomes imperative.

#### Regenerative surgery

The EFP guideline recommends periodontal regenerative surgery using barrier membranes or enamel matrix derivative with or without bone-derived grafts for deep residual pockets with intrabony defects (≥ 3 mm).^24^ Additionally, in regions characterized by a thin phenotype or inadequate keratinized gingiva (< 2 mm), there exists an elevated risk of gingival recessions during labial tooth movement. In such instances, as well as in cases with pre-existing gingival recessions, augmentation of bone and/or soft tissues (phenotype modification) is deemed necessary before commencing orthodontic intervention.^26,27^

### Orthodontic treatment

#### Perio-ortho joint evaluation/treatment planning

After systemic periodontal therapy, the endpoints of periodontal treatment have been achieved. Perio-ortho joint evaluation/treatment planning is necessary to determine further periodontal considerations for active orthodontic treatment. For example, in cases with elongated teeth, circumferential supracrestal fiberotomy before orthodontic intrusion may be performed to release the tension on the supra-alveolar fibers, thereby reducing marginal bone loss.^28^

#### Active orthodontic treatment

The foundational design principles and precautions for orthodontic treatment of patients with periodontal disease will be outlined in the subsequent two sections.

#### Orthodontic treatment towards completion

As the occlusal adjustment nears completion, implantation and/or restoration may be necessary in certain cases to achieve a stable occlusion.^29^

It is noteworthy that the treatment plan described above should not be considered dogma, but rather allows for some overlaps between steps. For example, dental implants may be performed before orthodontic treatment in cases where posterior anchorage is compromised. Additionally, the direction of orthodontic tooth movement may impact the timing of periodontal regenerative surgery. In mesially tilted molars with angular bony defects on the mesial surface, orthodontic uprighting of the molars can commence prior to regenerative surgery.

### Orthodontic retention and SPC

#### Perio-ortho final evaluation

The perio-ortho final treatment evaluation includes assessment of clinical attachment loss (CAL), probing pocket depth (PPD), BOP, and radiographic bone loss (RBL) by the periodontist and assessment of tooth alignment and occlusal stability by the orthodontist.^4^

#### Orthodontic retention and periodontal maintenance

Long-term orthodontic retention is crucial for preserving stable occlusion, while regular and consistent periodontal follow-up care is essential to prevent relapse and maintain periodontal health. The selection of retainers, scheduling of orthodontic recalls, and frequency of periodontal follow-ups should be tailored to individual patients’ periodontal conditions.

## Prerequisites And Design Of Orthodontic Treatment For Patients With Periodontal Disease

### Periodontal examination before orthodontic treatment

Before commencing orthodontic treatment, it is essential to perform a systematic and comprehensive assessment of the patient’s periodontal condition. This involves obtaining the patient’s medical history, conducting periodontal and occlusal examinations, and evaluating bone loss using radiographic data (Table 1).^14,30–32^ This assessment helps identify the risk factors, severity of periodontitis, and any secondary malocclusions caused by periodontal pathology, thereby facilitating the development of a collaborative periodontal-orthodontic treatment plan.

**Table 1:** Periodontal evaluation checklist for orthodontic treatment of patients with periodontal disease

Catogory		Items	Explaination in detail
**Patient history**		Chief complaint	Patient’s main complaint and expectations of treatment
		Dental history	Previous dental care and maintenance visits
		Medical history	Systematic diseases, medication
		Oral hygiene habits	Self-performed biofilm control
		Smoking	Assessment of smoking history and habits
**Clinical examination**	Periodontal status	Oral hygiene condition	Plaque index, gingival index, bleeding on probing
		Clinical attachment loss	Clinical attachment loss = probing pocket depth + gingival recession
		Gingival recession	Gingival recession and the presence or absence of interdental gingiva
		Periodontal soft phenotype	Thin or thick gingival phenotype, keratinized gingiva width
		Tooth mobility	Tooth mobility index (0-3°)
		Tooth anatomy	Square versus triangular tooth shape, amount of root exposure, presence and extension of dental abrasion, thickness of the incisal edges, crown-to-root length ratio, furcation involvement
	Occlusal evaluation	Trauma from occlusion	Fremitus, tooth migration, tooth mobility, attrition, tooth fracture, periodontal ligament space widening
		Pathologic migration of tooth	Extrusion, spacing, mesial tipping
**Radiographic examination**		Bone loss	Horizontal/vertical alveolar bone resorption (mild/moderate/severe)
		Periodontal bone phenotype	Thickness of the buccal and lingual bone plate evaluated by CBCT, bone dehiscence/fenestration
		Root	Root length in bone, root apex rounding, root resorption (mild/moderate/severe)

Note: Based on the publications of Han et al. ^14^, Milano et al. ^30^, Jepsen et al. ^31^, and Japsen et al. ^32^

### Communication with patients

Orthodontic treatment for patients with periodontal disease poses notable challenges, requiring thorough communication between healthcare providers and patients before treatment begins. Patients should be informed about the diagnosis of their condition, including its etiology and risk factors, the importance of compliance, potential risks and benefits of orthodontic treatment, and the complexity and variability of the treatment plan.^4,33,34^

- Orthodontists should first provide comprehensive information to patients regarding the causes and risk factors associated with their condition, aiming to foster compliance.
- Patient adherence is pivotal for successful periodontal-orthodontic combined therapy and the attainment of optimal treatment results. Patients should grasp the importance of self-performed oral hygiene and the adverse consequences of noncompliance. For example, the presence of fixed orthodontic appliances renders plaque removal more challenging, and poor compliance can accelerate attachment loss and periodontal tissue damage.
- While the benefits of orthodontic treatment for patients with periodontal disease are well-established, patients should also be aware of potential risks. For instance, individuals with thin gingival biotypes may encounter increased or exacerbated gingival recession following orthodontic treatment, resulting in heightened visibility of black triangles.
- Owing to the intricate nature of treatment, plans may require modifications during the course of therapy based on initial outcomes, patient cooperation, and new clinical findings.^35^

Thorough communication with patients before orthodontic treatment is essential for adequately preparing them for the time, effort, and financial commitment involved, thereby ensuring their understanding of treatment goals and potential challenges. Clarifying uncertainties during these discussions can significantly reduce the likelihood of medical disputes.

### The necessity and timing of orthodontic treatment

#### The necessity of orthodontic treatment

**Fig. 2:**
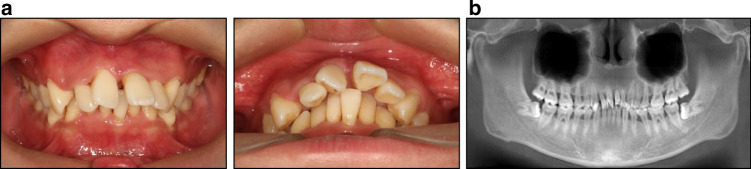
A 27-year-old male patient with traumatic occlusion. **a** Pathological tooth migration of the anterior maxillary teeth due to the loss of periodontal support is complicated by deepened overjet and overbite, spacing, and extrusion of maxillary incisors. **b** Radiographic image shows severe generalized horizontal bone loss with infrabony defects and furcation involvements

Orthodontic treatment plays a pivotal role in the multidisciplinary management of some patients with periodontitis, particularly those with traumatic occlusion. The decrease in alveolar bone height shifts the center of resistance (CR) apically, resulting in occlusal forces that progressively tip and extrude incisors due to increased shear stress on the alveolar bone. This initiates a detrimental cycle of tooth migration and shearing forces, leading to traumatic occlusion (Fig. 2). Concurrently, as teeth flare and overjet increases, the lower lip may be positioned behind the upper incisors during functional movements (e.g., swallowing, speech), exacerbating malocclusion. Moreover, bite collapse from posterior tooth loss can exacerbate the pathogenic migration of anterior teeth. Apart from tooth migration, traumatogenic forces can also disseminate dental biofilm and inflammatory exudates apically, exacerbating pocket depth and worsening attachment and bone loss. In cases of secondary malocclusion or deteriorating malocclusion due to periodontal tissue degradation, satisfactory outcomes cannot be achieved solely through periodontal and prosthodontic interventions.^36^ Consequently, orthodontic involvement becomes necessary to comprehensively address these challenges and achieve balanced, enduring outcomes.^37^

A recent study investigated the prevalence of pathologic tooth migration (PTM) in patients with stage III-IV periodontitis and their orthodontic treatment need based on PTM and anterior occlusal trauma.^7^ Among the 121 participants, PTM was prevalent in 74.4% of maxillary and 60.3% of mandibular anterior teeth. Additionally, orthodontic treatment need was identified in 66.1% of subjects. This study underscores the notable prevalence of PTM and the concomitant need for orthodontic care in patients with stage III-IV periodontitis. Furthermore, patients treated with combined periodontal-orthodontic treatment exhibited a notably lower rate of periodontal or orthodontic relapse (15%) compared to those solely receiving periodontal therapy (33%) 2 years post-treatment,^38^ indicating that achieving well-aligned occlusal relationships is beneficial for the long-term stability of treatment outcomes.

#### The timing of orthodontic treatment

There is a consensus that orthodontic tooth movement should commence only after effective control of periodontal inflammation and when the patient can maintain sufficient personal oral hygiene practices.^32^ The endpoints of periodontal therapy are achieved when there are (i) no periodontal pockets ≥ 5 mm with BOP, or (ii) no deep pockets ≥ 6 mm. Nonetheless, the ideal timing for initiating orthodontic treatment after active periodontal therapy has long been discussed.^21,22,39–43^ Considering the varying dynamics of periodontal healing following different therapeutic modalities,^44,45^ Pini Prato and Chambrone have proposed a comprehensive periodontal-orthodontic treatment model that recommends orthodontic treatment be commenced 3-6 months after non-surgical periodontal therapy, 6-9 months after non-regenerative surgery, and 12 months after regenerative procedures.^46^ Additionally, the Mario Aimetti research group suggested starting orthodontic treatment after the restoration of periodontal health and patient adherence to oral hygiene, typically occurring within 3 to 4 months after subgingival instrumentation or non-regenerative surgery, and 6 months after regenerative procedures.^11^ Another group proposed minimizing the interval between periodontal therapy and orthodontic treatment, suggesting initiation of orthodontic treatment immediately after non-surgical periodontal treatment and 2-4 weeks after surgical treatment.^14^ It should be noted that these recommendations are primarily empirical suggestions based on clinical experience of many years but not robust scientific evidence.

The timing for initiating orthodontic treatment after regenerative procedures in intra-bony defects has been a topic of debate. It is prudent to wait 6-12 months to reach the endpoint of regenerative therapy, avoiding disruption to periodontal healing. Several case reports and series, with follow-up periods extending beyond 3 years post-regenerative surgery, have demonstrated favorable periodontal outcomes with this delayed approach.^22,23,43,47^ However, some studies propose starting orthodontic tooth movement immediately or within 3 months post-regenerative surgery, suggesting no adverse effects and even potential stimulation of periodontal wound healing.^48–50^ Notably, a retrospective case series with stage IV periodontitis patients who underwent orthodontic treatment 3 months after regenerative surgery showed significant improvements after 12 months, with long-term maintenance observed for up to 4 years.^51^ A multicenter randomized clinical trial compared the periodontal outcomes of regenerative treatment of intrabony defects following early (after 4 weeks) and late (after 6 months) orthodontic treatment of individuals with adequate oral hygiene and inflammation control, finding comparable periodontal improvements (CAL gain, PPD reduction, and pocket closure) 12 months after periodontal surgery.^52^ Based on this clinical evidence, the EFP guideline suggests not waiting for a prolonged healing period post-regenerative intervention.^4^ The possibility and rationality of initiating orthodontic treatment early after regenerative surgery are of particular interest to both clinicians and patients, as this approach may shorten the overall treatment duration.

### The essentials in orthodontic design

#### Multidisciplinary, individualized, and compromised treatment plan

In cases of severe periodontitis, patients often face multiple challenges such as dentition defects, traumatic occlusion, and retrograde pulpitis, which significantly complicate treatment plans. A multidisciplinary diagnostic and therapeutic approach is essential to restore oral function and aesthetics effectively, thereby improving long-term outcomes. Furthermore, malocclusion resulting from periodontitis presents unique challenges that require personalized orthodontic interventions tailored to factors such as the varying resilience of periodontal tissues, overall health status, and age. It is crucial to avoid overly complex and prolonged orthodontic procedures, favoring instead practical treatment strategies and achievable objectives to ensure effective and efficient patient care.^33,34,53^

#### Biomechanics

**Fig. 3:**
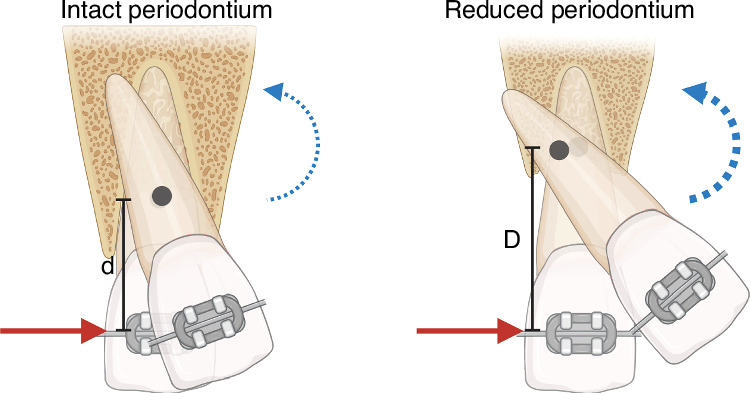
Schematic illustration showing the effect of a force when applied to the bracket of a tooth with either an intact or reduced periodontium

The primary challenge in managing patients with controlled periodontitis is the diminished vertical height of the alveolar bone, which disrupts the mechanics of tooth movement. As the bone height reduces, the CR shifts apically, leading to a higher moment-to-force ratio (M/F) (Fig. 3).^54^ Therefore, careful analysis of force direction, magnitude, and application point is crucial in designing the force system to prevent undesired tooth displacements.^12,13,55^

- The force level should be adjusted considering the reduced bone surface and periodontal ligament connected to the teeth. Stress and strain in the root increase with reduced periodontal ligament, it is therefore essential to minimize force magnitude to avoid indirect bone resorption and root resorption caused by hyalinization.
- With the apical movement of CR, uncontrolled inclination movements are more likely to occur, while achieving bodily movement becomes more challenging. To reduce the M/F ratio, it is recommended to bond orthodontic brackets as cervically as possible, ensuring they do not impede oral hygiene. In addition, clinical tooth crowns can be reshaped when necessary.
- Segmented mechanics are favored over continuous archwire mechanics because they allow for clear isolation of active and reactive forces/moments, thereby maximizing desired tooth movements while minimizing undesired ones. In the example of extruded and flared incisors, intrusive and retraction forces from an intrusion arch should be directed as close as possible to the CRs of the teeth to achieve controlled tipping and intrusion.^13^ A light (5-15 g per tooth) and continuous force is necessary to reduce periodontal ischemia and external apical root resorption.^56^ The reactive units of the intrusion arch can be directed onto either multiple connected posterior teeth or dental implants/TADs.

#### Orthodontic appliance

**Fig. 4:**
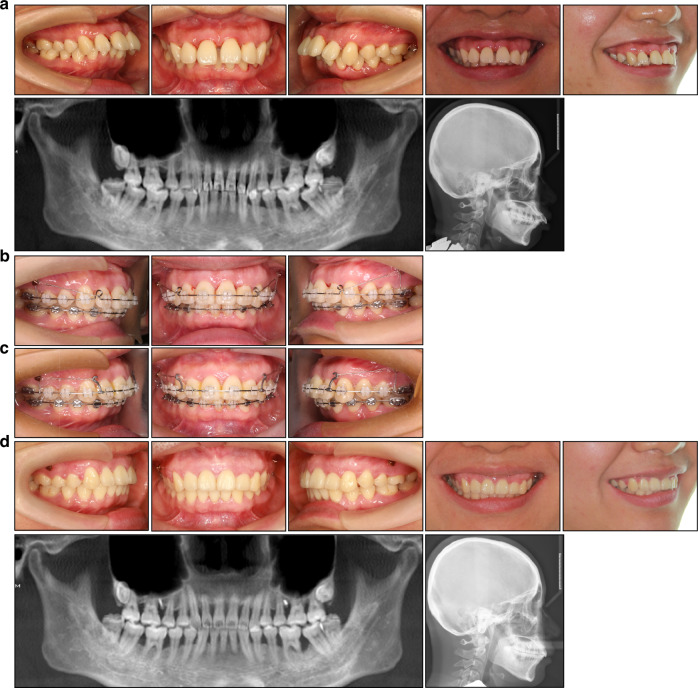
Treatment of a 38-year-old periodontally compromised female using the fixed appliance and TADs. **a** Pretreatment intraoral and facial view, panoramic radiograph, and lateral cephalogram. **b,** **c** After periodontal inflammation control, non-extraction orthodontic treatment with TADs was applied to achieve anterior teeth intrusion and retraction. **d** Posttreatment intraoral and facial view, panoramic radiograph, and lateral cephalogram. Positive functional and esthetic results were achieved

Most patients diagnosed with periodontitis are treated with fixed orthodontic appliances, which offer precise control over tooth movement, particularly in terms of rotation and intrusion (Fig. 4). However, fixed attachments and adhesive remnants tend to exacerbate plaque accumulation and shift both supra- and subgingival microflora towards more pathogenic species, posing a significant risk for periodontitis patients.^57,58^ To minimize these risks, it is advisable to utilize the simplest orthodontic system possible, thereby reducing plaque buildup and facilitating self-performed oral hygiene .

**Fig. 5:**
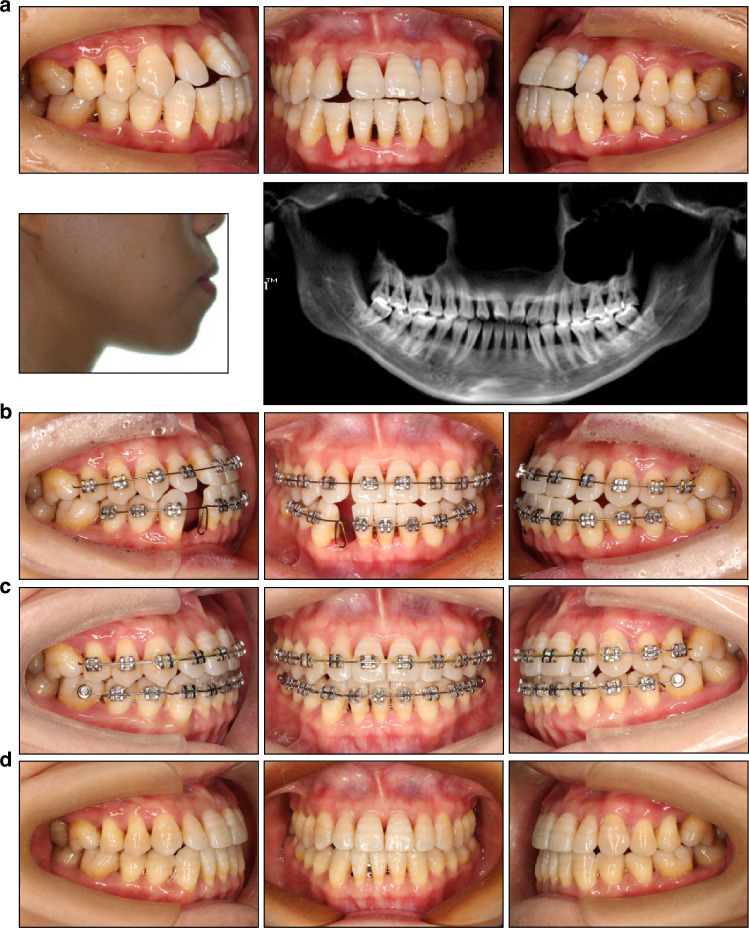
Treatment of a 34-year-old periodontally compromised female patient. **a** Pretreatment intraoral view, facial profile, and panoramic radiograph. The edge-to-edge bite, a full unit Class III molar relationship on the left side, and severe bone loss are evident. **b** The treatment goal is to eliminate occlusal trauma and improve occlusal contact, without excessively emphasizing on correcting the facial profile or molar relationships. Therefore, tooth 42, with the poorest periodontal condition, was selected for extraction. The first molars were strategically left unbonded to preserve their position and facilitate oral hygiene. **c** Lingual buttons were bonded on the mandibular first molars to close residual spaces. **d** Posttreatment intraoral view

- Employing bonded molar tubes instead of molar bands offers improved control over plaque accumulation at the gingival aspect of the appliance.^59^
- Steel ligatures are preferable as they significantly reduce plaque accumulation and microbial presence compared to elastomeric rings.^60^
- Meticulous removal of excess bonding material surrounding orthodontic brackets is crucial, as these adhesive remnants serve as prime sites for plaque buildup.^61^
- It is recommended to initially bond brackets only to the teeth requiring movement, delaying the placement of brackets on teeth not yet targeted for adjustment (Fig. 5). This staged approach helps maintain oral hygiene by avoiding the sudden introduction of numerous orthodontic attachments at the start of treatment.^34^

Clear aligners have gained increasing popularity among patients due to their aesthetic advantages over fixed orthodontic appliances.^62^ When multiple factors such as self-performed oral hygiene and constant clinical monitoring are considered, no definitive evidence has established the superiority of clear aligners over fixed appliances in maintaining periodontal health during orthodontic treatment.^63–65^ Although the clinical benefits and strategies of orthodontic treatment with fixed appliances in periodontitis patients have been extensively discussed, the utilization of clear aligners in such patients requires further exploration. Only a limited number of studies have reported orthodontic treatment using clear aligners in periodontitis patients, wherein improvements in periodontal parameters were achieved at the end of perio-ortho treatment.^66–70^ It is noteworthy that most of these studies are case reports/series involving patients with limited periodontal impairment, such as tooth mobility ≤ 1° and probing depth < 4 mm. Han et al. compared the effect of periodontal-orthodontic treatment with fixed and removable appliances on periodontal tissues in patients with periodontitis (probing depth = 2.58 mm ± 0.78 mm and bone loss = 3.53 mm ± 1.26 mm), where 19 patients were treated with fixed appliances and 17 with clear aligners.^71^ The groups did not exhibit significant differences in periodontal indices; however, the treatment duration was shorter in the fixed appliance group compared to the clear aligner group.

The severity of periodontal impairment should be taken into consideration for patients with periodontitis when using clear aligners.^11^ For instance, teeth with moderate mobility can be repositioned with fixed appliances and endure oblique forces without significant damage owing to stabilization provided by the orthodontic wire.^72^ However, in clear aligner therapy, teeth with moderate to severe mobility should not be subjected to the retentive forces exerted during the insertion and removal of orthodontic devices to avoid aggravation of tooth mobility, as proposed by Santos et al. ^11^ Due to the high demand for controlled orthodontic force for targeted tooth movement, the EFP guideline recommends using fixed rather than removable appliances in patients with severe periodontitis (stage IV or equivalent).^4^ Therefore, while clear aligners may be suitable for patients with mild to moderate periodontitis, their application in severe cases is currently not recommended, especially for less experienced clinicians.

#### Anchorages

Stable anchorage is essential for orthodontic treatment of patients with controlled periodontitis. As intrusion and retraction of extruded and protruded incisors are necessary for the majority of patients with periodontitis, posterior anchorage constitutes a critical aspect of anchorage design.^13^ However, it is difficult to obtain sufficient anchorage in patients with tooth loss and reduced alveolar bone support. Dental implants or temporary anchoring devices (TADs) are therefore commonly employed to reinforce anchorage due to their efficacy as optimal anchorage units.^73–75^

- In cases with posterior tooth loss, dental implants with provisional crowns should be placed before orthodontic treatment to serve as future anchorage units. The timing of orthodontic force application to the implant aligns with prosthetic loading, allowing for brackets to be bonded onto provisional crowns using standard procedures.^74^
- TADs can be positioned in various locations, including the interradicular space of the maxilla and mandible, palate, or distal to the second molar, which ensure enhanced safety for tooth movement and have become a clinical necessity.^76^ Orthodontic forces can be directly applied to miniscrews, known as direct anchorages, or TADs can be linked to natural teeth to augment anchorage, functioning as indirect anchorage within the biomechanical system.^77^
- Teeth intended for extraction can be retained to serve as anchorage and maintain occlusal support, thus deferring extraction until a later phase of treatment.

## Precautions Of Orthodontic Treatment For Patients With Periodontal Disease

### Monitor of orthodontic follow-up

During orthodontic follow-up appointments, orthodontists bear the responsibility of concurrently monitoring both tooth response to orthodontic forces and patients’ periodontal status.^34^ Evaluating the speed and type of tooth movement, as well as changes in occlusal relationships, is paramount to ensuring precise control over tooth movement. Equally crucial is the meticulous observation of patients’ compliance to self-performed oral hygiene practices and their periodontal conditions, including signs of gingival inflammation and increased tooth mobility. Patients should receive thorough instruction on proper tooth-brushing techniques upon the placement of braces and undergo close surveillance during subsequent visits. Failure to comprehensively assess periodontal health along with tooth movement may result in misjudgments regarding the speed of tooth movement and could lead to blindly escalating corrective forces. Applying excessive orthodontic force to teeth in the presence of inadequate inflammation control may not expedite tooth movement and could exacerbate damage to periodontal tissues.^78^ Thus, meticulous evaluation of periodontal conditions at each orthodontic visit are imperative for ensuring treatment efficacy.

### Regular periodontal maintenance

During orthodontic treatment, continuous professional periodontal maintenance is necessary. It is recommended to schedule regular scaling and root planing sessions based on the severity of periodontal damage and establish a supportive periodontal care plan. Typically, routine periodontal examinations and maintenance are advised every 3-6 months, including probing depth measurements, bleeding indices, and panoramic or periapical radiographs to assess alveolar bone remodeling.^33^ For patients with severe periodontitis, the interval between periodontal maintenance treatments can be shortened to align with the frequency of orthodontic follow-up visits (e.g., every 4–6 weeks).^4,34^

### Special considerations for periodontal-orthodontic treatment of children and adolescents

Special periodontal considerations are necessary for orthodontic patients in the mixed and early permanent dentitions, due to their unique periodontal developmental features and generally less consistent oral hygiene habits.^79^ During this stage, alveolar bone height varies, with a normal distance of 1-2 mm from the cemento-enamel junction to the alveolar bone crest in primary dentition. A greater distance, although still within normal limits, might be observed adjacent to shedding primary teeth or erupting permanent teeth. Vertical bone defects may emerge between erupting permanent and primary teeth, particularly in the premolar and first molar regions.^80^ These are often part of the normal tooth eruption process but should be monitored for future developments.^79^ Additionally, children and teenagers often exhibit shorter clinical crowns due to incomplete active or passive tooth eruption, resulting in greater gingival coverage.^81^ Consequently, orthodontic braces are typically positioned near the gingival margin. This anatomical factor, coupled with limited oral hygiene awareness among these individuals, predisposes them to gingivitis.^82^ In such instances, patients necessitate subgingival scaling and oral hygiene instruction. If gingival hyperplasia occurs, surgical intervention may be necessary following basic periodontal therapy.

### Management of periodontal complications during orthodontic treatment

During orthodontic treatment, periodontal complications may arise, such as gingivitis and the recurrence of periodontitis resulting from inadequate oral hygiene practices. Additionally, improper application of orthodontic forces may lead to secondary periodontal damage.^83,84^

In patients experiencing periodontitis recurrence, active orthodontic treatment should be halted upon detection of signs, and affected teeth should undergo passive maintenance while receiving appropriate periodontal treatment and reinforcement of oral hygiene practices.^4,85^ Ideally, all necessary procedures should be conducted during a single follow-up appointment, including the removal of archwires, periodontal treatment and maintenance overseen by a periodontist, followed by the reinsertion of archwires. However, if completing all steps in one session proves impractical or if periodontal treatment remains ongoing, simply removing archwires may not suffice. It is recommended to employ continuous ligations or passive archwires to preserve dental arch shape and stabilize teeth during the periodontal retreatment phase.^34^ Once periodontal health and stability have been restored, orthodontic treatment may be resumed.

Excessive or frequent orthodontic force can lead to considerable tooth mobility and root resorption.^86^ Furthermore, patients with diminished periodontal soft and bone tissue support are susceptible to bone dehiscence and fenestration when subjected to inappropriate forces.^87–89^ In such cases, orthodontic treatment should be temporarily suspended, and consultation with a periodontist may be necessary to determine subsequent treatment strategies.

### Tooth reshaping

Unevenly abraded teeth resulting from malpositioning and varying degrees of gingival recession in anterior teeth are common in adult orthodontic patients. These conditions contribute to inconsistent clinical crown lengths of the incisors, impairing facial and smile aesthetics.

- Orthodontists can camouflage the existing discrepancies in tooth anatomy through three-dimensional (3D) tooth reshaping.^90^ For teeth with elongated clinical crowns resulting from bone loss and gingival recession, adjusting the crown-root ratio via tooth reshaping not only improves the patient’s aesthetics but also benefits the mechanical functioning of anterior teeth.
- Sometimes, it is appropriate to conduct gross modification of the tooth anatomy prior to commencing orthodontic treatment; however, interproximal reshaping is deemed inappropriate at this stage.
- After 6-8 months of orthodontic treatment, reevaluation is needed with an interim panoramic radiograph.^30,90^ Special consideration should be given to the management of tooth contact points and black triangles, particularly in the upper incisors. Initially, if required, the length and incisal edge thickness of anterior teeth can be adjusted. Subsequently, adjustments to contact points to minimize or eliminate black triangles should be approached cautiously, avoiding excessive narrowing of the incisors or excessive proximity of the roots.
- When further reduction in crown width is not advised, minor medial tipping of central incisor roots may be considered to reduce interdental space and ensure adequate interdental gingival coverage.^91^

### Management of black triangles

Periodontitis can result in the loss of interdental papilla, often referred to as “black triangles”.^92^ Typically, one or more black triangles may develop after retraction and intrusion of migrated anterior teeth. Several factors contribute to the absence of papilla, including the distance from the alveolar ridge crest to the tooth contact point, root position and angle, tooth crown morphology, soft tissue volume, and interproximal cleaning habits.^93^ In addition to aesthetic and speech-related concerns, black triangles promote food retention, compromising periodontal health.

- Orthodontic intervention can address black triangles by repositioning teeth, closing diastemas, reducing the bone crest-contact point distance through interproximal stripping, and aligning roots with divergent angles.^92^
- In cases with advanced periodontal destruction, orthodontic treatment can be complemented with periodontal regenerative procedures like bone and soft tissue grafting to manage bone defects and aid in papilla reconstruction.^94,95^

## Orthodontic Retention And Periodontal Follow-Up

Post-orthodontic relapse poses both aesthetic and functional challenges that undermine treatment efficacy and patient satisfaction. Moreover, any instability in periodontal condition may induce occlusal instability, thereby compromising treatment outcomes.^96^ To ensure stable occlusion, long-term orthodontic retention and consistent periodontal care are essential.

### Orthodontic retention

Retention strategies utilizing either removable or fixed appliances are typically employed, taking into account factors such as the initial malocclusion, the type of tooth movements, patient preferences, and more importantly, the severity of periodontal disease.^97,98^

- For patients with well-controlled periodontal inflammation and alveolar bone absorption not exceeding one-third of the root, vacuum-formed removable retainers are recommended. It is crucial to note that proper blocking of undercuts in plaster models is essential during retainer fabrication to ensure adequate retention while minimizing undesired forces during wear and removal.^34^
- For patients with alveolar bone absorption extending to the middle third or up to the apical third of the root, lifelong retention with appropriately designed passive fixed retainers (e.g., multistranded stainless-steel wires) is preferred with or without additional removable retention.^4,12^
- The timing of orthodontic recalls should be tailored to individual patients based on their pre-existing occlusions and the severity of periodontitis.

The potential risks and problems of lingual fixed retainers should be acknowledged by both orthodontists and patients.^12^ These retainers must be meticulously adjusted to accommodate the tooth morphology and bonded with precision to prevent the formation of adhesive remnants, which can act as sites for plaque accumulation.^99,100^ Additionally, while lingual fixed retainers prove effective in preventing/reducing tooth relapse, some inadvertent movements of the teeth can occur due to the distortion of the wire, ranging from minor tooth displacement to gingival recession, or even tooth vitality loss.^101–103^ Such adverse effects can be particularly problematic for patients with severe periodontitis. Thus, it is imperative to inform these patients of such potential risks and ensure regular monitoring to promptly detect any occurrences. Furthermore, the median survival time of lingual retainers is approximately 4.5 years, primarily attributable to breakage or accidental debonding.^104^ Recent research indicates that individuals with severe periodontitis exhibit a higher incidence of retainer failure compared to those with milder forms of the condition,^105^ underscoring the necessity for frequent follow-up appointments to assess retainer integrity in severe periodontitis cases.

**Fig. 6:**
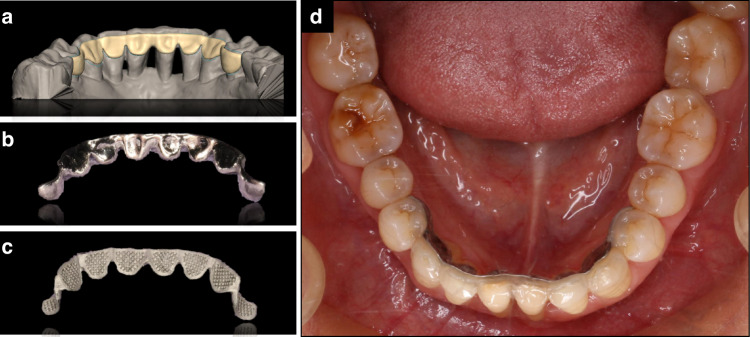
Utility of 3D-printed lingual retainer in an orthodontic patient with controlled periodontitis. **a**–**c** Digital design **a** and photographs of the 3D-printed mandibular lingual retainer with smooth lingual surface **b** and mesh base for bonding **c**. **d** Intraoral view of the retainer bonded to teeth 34–44

It is noteworthy that the advent of 3D printing has opened new visions in orthodontics,^106–108^ with 3D printed lingual retainers (Fig. 6) emerging as a promising alternative to conventional fixed retainers.^109^ Fabricated from materials such as resin, metal, and polylactic acid, these innovative retainers offer streamlined clinical workflows and increased convenience for both patients and practitioners. Owing to the precision and customization of 3D printing technology, these retainers can more accurately conform to the patient’s dental morphology. The smooth lingual surface of the retainer is beneficial for oral hygiene, reducing plaque builup and sulcus formation. In vitro studies have demonstrated better mechanical and bonding strength of 3D-printed retainers compared to traditional counterparts.^110,111^ A recent prospective randomized clinical trial observed significant advantages of 3D-printed retainers over traditional fixed ones, including improved periodontal health, reduced relapse rate, and fewer instances of failure during a 6-month follow-up period.^112^ Nevertheless, further long-term clinical trials are warranted to thoroughly evaluate the clinical efficacy of 3D-printed retainers.

### Long-term periodontal follow-up

The frequency of periodontal consultations should be tailored to each patient’s periodontal status and risk of recurrence, typically involving initial follow-ups every 3 months for the first year, followed by biannual visits in subsequent years.^9^ In the event of periodontitis recurrence, prompt diagnosis and treatment are imperative. Moreover, compliant patients who adhere to self-performed oral hygiene practices and lead healthy lifestyles exhibit lower rates of periodontitis recurrence and tooth loss compared to non-compliant individuals,^113,114^ underscoring the significant contribution of patient adherence to long-term periodontal health.

## Clinical Evidence On The Effects Of Orthodontic Treatment In Patients With Periodontal Disease

### Effects on periodontal parameters

Clinical evidence suggests that orthodontic movement in patients with well-controlled periodontitis does not significantly impact periodontal outcomes, gingival inflammation, gingival recession, or root resorption.^115^ A recently published systematic review, encompassing 40 studies involving a total of 1608 Stage IV periodontitis patients, revealed that combined periodontal-orthodontic treatment offers significant advantages over periodontal treatment alone, including greater CAL gains (-0.55 mm to -0.14 mm), greater PPD reduction (-1.07 mm to -0.27 mm), greater RBL improvement (-0.11 mm to -0.01 mm), fewer cases with III° tooth mobility (0.08 to 0.52), less treatment failure (0.05 to 0.42), and improved patient-reported outcomes (PROs).^12^ Therefore, there has been substantial evidence supporting orthodontic treatment of patients with controlled periodontitis when clinically indicated.

### Specific orthodontic movements

#### Extrusion

Orthodontic extrusion of teeth has been reported to increase alveolar ridge height and reduce periodontal pockets in patients with periodontitis.^116^ This process involves the coronal movement of gingiva attached to the alveolar bone, thereby augmenting the width of keratinized gingiva and enhancing gingival aesthetics.^116,117^ In individuals with healthy periodontium, tooth extrusion induces concurrent coronal movement of the gingival margin and mucogingival junction in 80% and 52.5% of cases, respectively.^118^ For teeth with a hopeless prognosis, orthodontic extrusion before implant placement may serve as an alternative to bone augmentation procedures, which improves both hard and soft tissue morphology in the implant recipient site and offers favorable restorative outcomes.^119^ A systematic review included clinical studies on orthodontic extrusion of hopeless maxillary anterior teeth and found that all these studies reported consistent improvements in bone availability at the implant recipient site, although the majority of these studies were case reports or series.^120^ It is advised to apply gentle, constant forces (15 g for anterior teeth and 50 g for posterior teeth) and buccal root torque to increase buccolingual bone thickness, maintaining a slow and steady rate (not exceeding 2.0 mm per month). Furthermore, after successful extrusion, a stabilization period of 3 to 6 months is recommended prior to tooth extraction.^121^

#### Intrusion

While the phenomenon of bone apposition after gradual extrusion is extensively documented, the impact of orthodontic intrusion on periodontal tissues remains controversitial. Orthodontic intrusion may relocate supra-gingival plaque to the subgingival region, potentially exacerbating periodontal bone loss and the development of infrabony periodontal pockets.^122,123^ Nevertheless, meticulous oral hygiene can mitigate this risk, and orthodontic intrusion coupled with thorough periodontal treatment has shown clinical efficacy in improving the health of compromised periodontium, particularly in the anterior teeth.^39,41,124^ Melsen et al. treated 30 periodontitis patients with elongated incisors by incisor intrusion and found reduced clinical crown length (0.5 to 1.0 mm reduction) and decreased distance between the marginal bone level to the cementoenamel junction.^56^ Cardaropoli and Corrente et al. treated 10 patients suffering from severe periodontitis with open flap surgery and then orthodontic treatment using the segmented arch technique 7-10 days after surgery.^41,124^ An average of 2.1 mm intrusion, significant CAL gain, and PPD reduction were achieved after the combined treatment. In a separate study, Cao et al. explored the efficacy of orthodontic intrusion coupled with circumferential supracrestal fiberotomy in 14 periodontitis patients with 56 elongated incisors exhibiting horizontal bone loss.^28^ Following initial periodontal treatment and circumferential supracrestal fiberotomy, the orthodontic intrusion was performed utilizing a utility arch technique, applying forces ranging from 10-15 g per tooth. The results showed a significant reduction in marginal bone loss reduction and an increase in CAL and labial bone thickness. These studies suggest that with adequate control of periodontal inflammation and the application of light forces, orthodontic intrusion can be beneficial for periodontal tissues.

#### Molar uprighting

Tilted molars are common complications in patients with periodontitis following posterior tooth loss, often resulting in bite collapse and diminished vertical dimension. There have been limited studies reporting the effects of molar uprighting with orthodontic appliances on periodontal parameters. A retrospective cohort study involving 18 patients revealed that pre-prosthetic uprighting of 30 mesially tipped lower molars after successful non-surgical periodontal therapy led to substantial PPD reduction and CAL gain on the mesial and lingual aspects.^125^ Another study of 22 patients found no differences in alveolar bone height and gingival inflammation scores between uprighted molars and control teeth, while the pockets mesial to the uprighted molars exhibited shallower depths compared to those of control teeth.^126^ However, Brown et al. documented mesial marginal bone loss ranging from 0.5 to 1 mm as a consequence of molar up-righting.^127^ When a tilted molar presents furcation involvement, orthodontic uprighting may worsen the periodontal condition, unless stringent measures are implemented to control any periodontal inflammation.^128^ Given the absence of robust evidence on the potential effects of molar uprighting on periodontal prognosis, conclusive recommendations regarding the advisability of orthodontic intervention for periodontal patients remain elusive.^129^

#### Movement in edentulous areas

Orthodontic movement of teeth with a reduced but healthy periodontium into edentulous regions is usually feasible with minimal bone loss, granted that the movement aligns parallel to the ridge and employs gentle orthodontic forces.^130^ However, even in optimal scenarios, there remains a risk of attachment and bone support loss.^131^ In edentulous regions where buccolingual width is notably diminished following prolonged tooth loss, orthodontic movement through cortical bone may be impeded, potentially leading to buccal and lingual bone dehiscences. To mitigate these risks, it is suggested to augment alveolar bone width through bone augmentation prior to orthodontic treatment.^132^

#### Orthodontic movement of teeth associated with augmented intrabony defects

Periodontal regenerative procedures are frequently employed in the management of chronic periodontitis, especially in cases involving angular bony defects.^44,133^ The research focus of orthodontic treatment of patients who have undergone periodontal regenerative surgeries includes the quality and stability of tooth movements through the regenerated periodontal tissues, the optimal timing for starting orthodontic treatment after regenerative therapy, as well as the effects of combined treatment on clinical periodontal parameters.^130^

The optimal timing for initiating orthodontic treatment after surgery has been previously discussed, and the remaining two issues have been investigated in experimental studies and validated in clinical studies. Na et al. conducted a case series study examining histologic biopsy samples from human teeth treated with guided tissue regeneration (using bio-oss and collagen membrane) alongside orthodontic movement, either directed away from or into the augmented defect.^134^ Their findings revealed a higher degree of new bone formation in sites subjected to both tensile and pressure forces compared to those treated solely by regenerative procedures. Additionally, the volume occupied by graft particles decreased in sites where a combined approach was implemented. The increased bone formation within intrabony defects following combined therapy aligns with findings from a histological study in pigs, wherein alloplastic materials were utilized for regenerative therapy.^135^

Clinical studies have consistently demonstrated improved periodontal parameters following combined periodontal regenerative therapy and orthodontic treatment. For example, in 526 defects (across 48 patients) subjected to orthodontic movement following regenerative surgery, Tietmann et al. found significant PPD reduction from baseline (6.00 mm ± 2.09 mm) to 1 year (3.45 mm ± 1.2 mm) and 2-4 years (3.12 mm ± 1.36 mm), as well as pocket closure (PPD ≤ 4 mm) in 87% of defects.^51^ Additionally, bone level gains were 4.67 mm ± 2.5 mm and 4.85 mm ± 2.55 mm after 1 year and up to 4 years, respectively. The authors compared the results with another study where patients had similar baseline characteristics and were treated with the same generative protocol but without the need for orthodontic treatment.^136^ Notably, patients receiving the combined treatment demonstrated greater improvement in bone level gain and PPD reduction compared to those treated solely with periodontal regenerative surgery. The researchers postulated that orthodontic tooth movement may stimulate the healing process and enhance the efficacy of periodontal regenerative procedures.

### Patient-reported outcomes

PROs denote reports on patients’ oral health status provided directly by the patients themselves, without interpretation by clinicians or others.^137^ These assessments are instrumental in gauging the impact of diseases or interventions on patients. While evidence concerning PROs following periodontal-orthodontic combined therapy remains limited, recent research has indicated significant enhancements in PROs measures related to esthetics and masticatory function.^138^ Notably, most patients who expressed dissatisfaction with their smiles experienced complete satisfaction following completion of the periodontal-orthodontic treatment.^138^ Despite the existing evidence from this retrospective study, one of the important future directions for research should be a thorough investigation of PROs in multi-center, large-scale samples of periodontal-orthodontic combined therapy through controlled studies. This approach can not only enhance the consistency of research but also improve its external validity, thereby more accurately assessing treatment outcomes and better meeting the needs and preferences of patients.

## Conclusions and Expectations

With the global prevalence of periodontal disease and the rising demand for orthodontic treatment, the integration of periodontal and orthodontic therapies has emerged as a significant focus in the field of oral medicine. This expert consensus comprehensively addresses the indications, objectives, procedures, and considerations associated with orthodontic treatment of periodontal patients, with the aim of offering clear and practical guidance for clinical practitioners. Emphasizing the importance of thorough diagnostic assessment in evaluating the extent of periodontal disease and primary/secondary malocclusions, the consensus underscores the necessity of sequential periodontal-orthodontic interventions, along with patient compliance throughout the entire process, as pivotal elements for achieving favorable treatment outcomes. Additionally, long-term retention and periodontal follow-up are deemed essential to ensuring treatment success.

Future research directions include further clinical investigations into the long-term effectiveness of orthodontic intrusion, molar uprighting, PROs, and 3D-printed fixed retainers in periodontal patients. Moreover, with advancements in medical technology, tissue engineering strategies for periodontal regeneration are anticipated to integrate into orthodontic care for patients with bone and/or soft tissue defects. These strategies involve the use of stem cells, growth factors, and other bioactive substances within bioactive scaffolds to promote the regeneration and repair of periodontal tissues.^139,140^ The implementation of such methods may enhance the healing capacity of periodontal tissues or provide stronger support for tooth stability after orthodontic treatment.
